# Estimation and consequences of direct-maternal genetic and environmental covariances in models for genetic evaluation in broilers

**DOI:** 10.1186/s12711-023-00829-8

**Published:** 2023-08-07

**Authors:** Hélène Romé, Thinh T. Chu, Danye Marois, Chyong-Huoy Huang, Per Madsen, Just Jensen

**Affiliations:** 1 Center for Quantitative Genetics and Genomics, Aarhus University, 8830 Tjele, Denmark; 2 Faculty of Animal Science, Vietnam National University of Agriculture, Gia Lam, Hanoi, Vietnam; 3 Cobb-Vantress Inc, Siloam Springs, AR 72761-1030 USA

## Abstract

**Background:**

Maternal effects influence juvenile traits such as body weight and early growth in broilers. Ignoring significant maternal effects leads to reduced accuracy and inflated predicted breeding values. Including genetic and environmental direct-maternal covariances into prediction models in broilers can increase the accuracy and limit inflation of predicted breeding values better than simply adding maternal effects to the model. To test this hypothesis, we applied a model accounting for direct-maternal genetic covariance and direct-maternal environmental covariance to estimate breeding values.

**Results:**

This model, and simplified versions of it, were tested using simulated broiler populations and then was applied to a large broiler population for validation. The real population analyzed consisted of a commercial line of broilers, for which body weight at a common slaughter age was recorded for 41 selection rounds. The direct-maternal genetic covariance was negative whereas the direct-maternal environmental covariance was positive. Simulated populations were created to mimic the real population. The predictive ability of the models was assessed by cross-validation, where the validation birds were all from the last five selection rounds. Accuracy of prediction was defined as the correlation between the predicted breeding values estimated without the phenotypic records of the validation population and a predictor. The predictors were the breeding values estimated using all the phenotypic information and the phenotypes corrected for the fixed effects, and for the simulated data, the true breeding values. In the real data, adding the environmental covariance, with or without also adding the genetic covariance, increased the accuracy, or reduced deflation of breeding values compared with a model not including dam–offspring covariance. Nevertheless, in the simulated data, reduction in the inflation of breeding values was possible and was associated with a gain in accuracy of up to 6% compared with a model not including both forms of direct-maternal covariance.

**Conclusions:**

In this paper, we propose a simple approach to estimate the environmental direct-maternal covariance using standard software for REML analysis. The genetic covariance between dam and offspring was negative whereas the corresponding environmental covariance was positive. Considering both covariances in models for genetic evaluation increased the accuracy of predicted breeding values.

**Supplementary Information:**

The online version contains supplementary material available at 10.1186/s12711-023-00829-8.

## Background

**Fig. 1:**
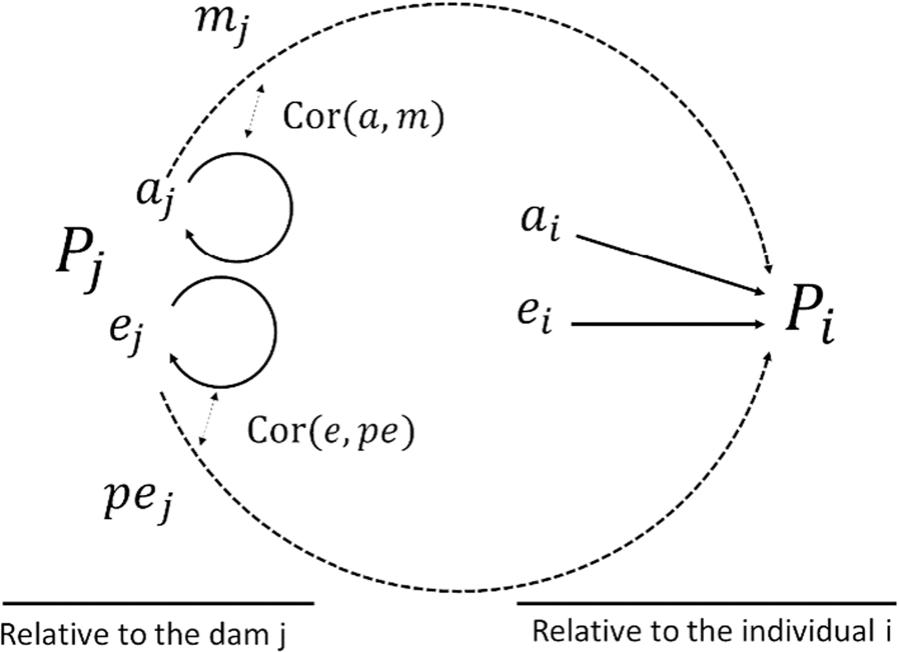
Diagram describing a phenotype influenced by maternal effects (adapted from Willham 1963)

Juvenile traits are influenced by maternal effects in many species [1–3]. The maternal effect can be divided into two major categories, general fixed effects and animal-specific effects, which are usually analyzed as random effects. The fixed effects will affect all the offspring of all dams belonging to the same class or level of the fixed effect in a common way. Such effects could be the age of the dam, the season of hatching, etc. Animal-specific effects, which include the genetic and permanent environmental maternal effects, will affect only the offspring of a given dam. Ignoring maternal effects might lead to bias in the prediction of breeding values and a reduction in prediction accuracy [4]. Indeed, if the maternal genetic effect is ignored, then part of this effect is captured by the direct additive genetic effect and therefore it will affect the predicted breeding values. This could lead, for example, to an overestimation of the genetic trend. For maternally-influenced traits, Willham [5] developed the maternal animal model where the phenotype for a given individual is equal to the sum of the phenotypic individual or direct effect and a phenotypic maternal effect. Therefore, the well-known equation ${P}_{i}={G}_{i}+ {E}_{i}$ becomes ${P}_{i}={G}_{i}+ {E}_{i}+ {G}_{j}+ {E}_{j}$, where ${P}_{i}$, ${G}_{i}$ and ${E}_{i}$ are the phenotype, the direct additive genetic effect and the direct environmental effect, respectively, for individual $i$ and ${G}_{j}$ and ${E}_{j}$ are the additive genetic effect and the environmental effect respectively of the dam $j$ as expressed in the phenotype of individual $i$. With such a model, a covariance structure between the direct and the maternal effects may arise. Indeed, usually the direct additive genetic effect of a dam is correlated to its maternal genetic effect, which is expressed in its offspring (Fig. 1). In our paper, we refer to this covariance as the Cor(a, m) or the direct-maternal genetic covariance. Similarly, with the environmental direct-maternal effect, the covariance between the residual (environmental) effect on the dam when it grew up (referred to as ${e}_{j}$ in Fig. 1) and the permanent environmental effects of the dam on its offspring (referred to as ${pe}_{j}$ in Fig. 1) needs to be considered. In our paper, we refer to this covariance as the Cor(e, pe) or the direct-maternal environmental covariance.

${P}_{i}$, ${a}_{i}$ and ${e}_{i}$ are the phenotype, the direct additive genetic effect and the direct environmental effect, respectively, for individual *i*. ${P}_{j}$, ${a}_{j}$ and ${e}_{j}$ are the phenotype, the direct additive genetic effect and the direct environmental effect, respectively, of dam *j*. ${m}_{j}$ and ${pe}_{j}$ are the maternal genetic effect and the maternal permanent environmental effect, respectively, as expressed in the phenotype of individual *i*.

Fitting a model that considers the direct-maternal genetic covariance (denoted as Cor(a, m)) using common breeding software is straightforward, and thus, the relation between the direct additive genetic effect and the maternal genetic effect has been widely discussed and investigated for decades [1, 2, 6, 7]. Highly negative direct-maternal genetic correlations have often been reported [1, 2, 7]. It has been proposed that a negative direct-maternal genetic covariance was due to a highly negative environmental covariance, which is commonly ignored in the analyses conducted to date [7, 8] or due to the collinearity of the direct and the maternal genetic effects and the negative sampling covariance between them [5]. However, except in special cases, there is no reason to assume that the direct-maternal genetic covariance should be positive [5, 9]. To investigate the reason for the negative correlation, different approaches to correct for the environmental direct-maternal correlation (denoted as Cor(e, pe)) have been developed. One way is to include a regression on the phenotype of the dam [1, 7]. With these methods, the maternal effects of several generations will be cumulated. Indeed, by adding the phenotype of the dam in the model, not only the direct effect of the dam is added but also that from its mother and so on [9]. Thus, compared with the maternal animal model, those methods raise a difficulty regarding the interpretation of the estimates. In 1997, Koerhuis and Thompson [1] tested different models based on those proposed by Willham [4] and Falconer [8]. One of the models accounted for both the direct-maternal genetic and the environmental covariance and was fitted using the restricted maximum likelihood (REML) methodology. Quintanilla et al. [10] proposed an extension of the maternal animal model that focused on the correlation of the genetic effect with the environmental maternal effect across generations. In this case, the maternal genetic effect of a dam will be correlated with the maternal genetic effects of its daughters and so on. Munilla and Cantet [11] proposed an approach to estimate the environmental direct-maternal covariance, i.e., a covariance between the environmental effect of the dam and the maternal environmental effect of its offspring, and estimated this correlation using a Bayesian procedure. However, because of the very tedious computational requirements of this method, it might be complicated to apply to large datasets.

To determine which model has the best fit to the data, the likelihood ratio test (LRT) is often used, and to evaluate the accuracy of prediction of breeding values, cross-validation (CV) is a common strategy. A common CV strategy is to train the models on a subset of the data, the training dataset, and then validate it on another subset, the validation dataset. A way to do this in animal breeding is to mask the phenotypes for some of the individuals, often the last few generations, and their breeding values are predicted based on the information of their relatives in the training population. Then, the accuracy of the prediction of breeding values based on pedigree information only (before the phenotypic record is obtained) is estimated as the correlation between the predicted breeding values and the phenotype corrected for fixed effects.

Legarra and Reverter [12] proposed an approach to estimate the relative change in population accuracy and bias using the breeding values that are estimated using the full dataset and reduced dataset. This method was developed to allow a comparison of different models in complex cases including, for example, maternal effects. However, in the presence of indirect genetic effects, two problems arose for estimating the accuracy of prediction of the model. The first problem is that there is a change in the definition of the breeding values depending on whether the models include or not the indirect genetic effects. Indeed, if a model including maternal effects is compared with one that ignores them, then the breeding values for the direct genetic effects obtained with the two models will have different definitions of the breeding values. In the first case, the breeding value for the direct genetic effects will reflect the additive genetic part only, whereas in the second case the breeding value will include the breeding value for the direct effects but also some of the maternal genetic effect. In this regards, the method proposed by Legarra and Reverter [12] encounters a problem to detect the biases in the models. The other problem is that the corrected phenotype might also be affected by indirect genetic effects. Therefore, the classical CV strategy may also encounter a problem to detect the biases in the models. Several approaches have been proposed to solve these issues, such as estimating the correlation between paternal half-sibs that do not share common maternal effects [13], such that the breeding values and corrected phenotype are freed of the maternal effects.

The accuracy of the estimates of the maternal genetic variance and of the covariance between direct and maternal genetic effects depends highly on the data structure, the number of dams having their own performance and on the number of progeny per dam [14, 15]. Broilers are a very relevant case for estimating the genetic and environmental direct-maternal covariance because in poultry a dam can produce a large number of progeny and also because maternal effects influence body weight (BW) in broilers [1, 13, 16, 17], although the maternal effects in broilers usually decrease with age compared to beef cattle, for which such effects can be long lasting.

The aims of this paper were to: (1) propose an extension to common standard REML methods to estimate the environmental direct-maternal covariance, by applying models that are similar to that proposed by Koerhuis and Thompson [1], using standard software for genetic analysis; (2) compare the performance of different CV methods in the presence of maternal effects; (3) validate our approach by estimating the environmental direct-maternal correlation in both real and simulation data; and (4) investigate the impact of adding genetic and direct-maternal environmental covariance into a prediction model on the accuracy and inflation of the predicted breeding values.

## Methods

### Birds

The commercial broiler data were provided by Cobb-Vantress Inc. (Siloam Springs, AR, USA), and the trait analyzed was BW measured in g at a fixed age after birds were hatched. The pedigree consisted of 407,473 birds, and phenotypic records were collected on 358,196 birds from 41 selection rounds (SR). At each SR, parents were selected based on their breeding values estimated with a pedigree-best linear unbiased prediction (BLUP) model. Individuals were mated following a hierarchical mating system where one dam is mated to only one sire, whereas a sire is mated to multiple dams. Table 1 provides an overview of the data.

**Table 1:** Description of the real data for body weight (BW) per sex

	Number of observations	Mean of BW (g)	SD of BW (g)
Females	179,897	1859	215
Males	178,299	2066	278

### Estimation of the variance components and breeding values

The animal model for traits that are affected by maternal effects was developed by Willham in 1963 [5]. This model assumes that the phenotype of an individual is influenced by its additive genetic effect and its environmental effect but also by the genetic background of the dam (genetic maternal effect, ${m}_{j}$ in Fig. 1) and by a permanent environmental effect of the dam that affects its offspring. Because the dam is itself an individual, this implies the existence of direct-maternal genetic (Cor(a,m)) and environmental (Cor(e,pe)) covariances. This was further described by Bijma in 2006 [9]. The idea behind the direct-maternal genetic covariance is that the additive genetic effect of the dam as an individual (i.e., observed on itself; ${a}_{j}$) is correlated with its maternal genetic effect (${m}_{j}$) observed on its offspring. The same goes for the direct-maternal environmental covariance where the environmental residual effects on the dam (${e}_{j}$ are correlated with the environmental effect of the dam on its offspring (${pe}_{j}$).

In broilers, sexual dimorphism affects the variances of BW, thus we considered that the BW in females and males were different but correlated traits. For the real data, five bivariate models were tested. The general model was defined as:

$$\left[\begin{array}{c}{\mathbf{B}\mathbf{W}}_{\mathbf{F}}\\ {\mathbf{B}\mathbf{W}}_{\mathbf{M}}\end{array}\right]= \left[\begin{array}{cc}{\mathbf{X}}_{\mathrm{F}}& {\mathbf{0}}\\ {\mathbf{0}}& {\mathbf{X}}_{\mathrm{M}}\end{array}\right]\left[\begin{array}{c}{\mathbf{b}}_{\mathrm{F}}\\ {\mathbf{b}}_{\mathrm{M}}\end{array}\right]+ \left[\begin{array}{cc}{\mathbf{Z}}_{\mathrm{F}}& {\mathbf{0}}\\ {\mathbf{0}}& {\mathbf{Z}}_{\mathrm{M}}\end{array}\begin{array}{cc}{\mathbf{W}}_{\mathrm{F}}& {\mathbf{0}}\\ {\mathbf{0}}& {\mathbf{W}}_{\mathrm{M}}\end{array}\right]\left[\begin{array}{c}{\mathbf{a}}_{\mathrm{F}}\\ {\mathbf{a}}_{\mathrm{M}}\\ \begin{array}{c}{\mathbf{m}}_{\mathrm{F}}\\ {\mathbf{m}}_{\mathrm{M}}\end{array}\end{array}\right]+\left[\begin{array}{cc}{\mathbf{B}}_{\mathrm{F}}& {\mathbf{0}}\\ {\mathbf{0}}& {\mathbf{B}}_{\mathrm{M}}\end{array}\begin{array}{cc}{\mathbf{W}}_{\mathrm{F}}& {\mathbf{0}}\\ {\mathbf{0}}& {\mathbf{W}}_{\mathrm{M}}\end{array}\right]\left[\begin{array}{c}{\mathbf{e}}_{\mathrm{F}}\\ {\mathbf{e}}_{\mathrm{M}}\\ \begin{array}{c}{\mathbf{p}\mathbf{e}}_{\mathrm{F}}\\ {\mathbf{p}\mathbf{e}}_{\mathrm{M}}\end{array}\end{array}\right],$$

where $\mathbf{X}$, $\mathbf{Z}$, $\mathbf{W}$ and $\mathbf{B}$ are the incidence matrices for the fixed effect, the additive genetic effect, the maternal effect and the environmental effect, respectively. $\mathbf{b}$ is the vector of the fixed effect of selection-round-hatch of the dam in interaction with the SR of the chick and the dam age in weekly classes. $\mathbf{a}$, $\mathbf{m}$, $\mathbf{p}\mathbf{e}$ and $\mathbf{e}$ are the vectors of the additive genetic effect, the maternal additive genetic effect, the permanent environmental maternal effect and the residual environmental effect, respectively. The subscripts $\mathrm{F}$ and $\mathrm{M}$ define the sexes for which the matrices and vectors are defined. Observe that in this model, the usual residual effects are now defined as ${\mathbf{e}}_{\mathrm{F}}$ and ${\mathbf{e}}_{\mathrm{M}}$ and that proper design matrices are defined for this residual as well. To be able to fit a model with the direct-maternal environmental covariance using standard software, we add a dummy residual that needs to be restricted to a constant. This constant value can be any value smaller than the true residual. In our study, we fixed this value to 1. We will refer to this fixed residual as the dummy residual. The true residual of the model is then equal to the sum of the estimated residual and the dummy residual.

The first model, “Moda,” ignores the genetic maternal variance and assumes that both the direct-maternal genetic and environmental covariances are null.

$$\left[\begin{array}{c}{\mathbf{a}}_{\mathbf{F}}\\ {\mathbf{a}}_{\mathbf{M}}\\ \begin{array}{c}{\mathbf{m}}_{\mathbf{F}}\\ {\mathbf{m}}_{\mathbf{M}}\end{array}\end{array}\right]\sim MVN\left[0, \mathbf{A} \otimes \left(\begin{array}{c}\begin{array}{c}{\sigma }_{{a}_{F}}^{2}\\ {\sigma }_{{a}_{F}{a}_{M}}\end{array}\\ \begin{array}{c}0\\ 0\end{array}\end{array} \begin{array}{c}\begin{array}{c}{\sigma }_{{a}_{F}{a}_{M}}\\ {\sigma }_{{a}_{M}}^{2}\end{array}\\ \begin{array}{c}0\\ 0\end{array}\end{array} \begin{array}{c}\begin{array}{c}0\\ 0\end{array}\\ \begin{array}{c}0\\ 0\end{array}\end{array} \begin{array}{c}\begin{array}{c}0\\ 0\end{array}\\ \begin{array}{c}0\\ 0\end{array}\end{array}\right)\right],$$

$$\left[\begin{array}{c}{\mathbf{e}}_{\mathbf{F}}\\ {\mathbf{e}}_{\mathbf{M}}\\ \begin{array}{c}{\mathbf{p}\mathbf{e}}_{\mathbf{F}}\\ {\mathbf{p}\mathbf{e}}_{\mathbf{M}}\end{array}\end{array}\right]\sim MVN\left[0, \mathbf{I} \otimes \left(\begin{array}{c}\begin{array}{c}{\sigma }_{{e}_{F}}^{2}\\ 0\end{array}\\ \begin{array}{c}0\\ 0\end{array}\end{array} \begin{array}{c}\begin{array}{c}0\\ {\sigma }_{{e}_{M}}^{2}\end{array}\\ \begin{array}{c}0\\ 0\end{array}\end{array} \begin{array}{c}\begin{array}{c}0\\ 0\end{array}\\ \begin{array}{c}{\sigma }_{{pe}_{F}}^{2}\\ {\sigma }_{{pe}_{F}{pe}_{M}}\end{array}\end{array} \begin{array}{c}\begin{array}{c}0\\ 0\end{array}\\ \begin{array}{c}{\sigma }_{{pe}_{F}{pe}_{M}}\\ {\sigma }_{p{e}_{M}}^{2}\end{array}\end{array}\right)\right].$$

Note that the residuals for the males were independent of the residuals for the females since they belong to records on different individuals. This model was included because it has been the commonly used model in several broiler-breeding programs.

For each of the following models, different assumptions on the variance–covariance structure were made as presented below. $\mathbf{A}$ is the pedigree relationship matrix and $\mathbf{I}$ is an identity matrix. The variance of the dummy residual remained constant for all the models.

The second model, “Modam,” assumes that the genetic and environmental covariances are null, but a maternal genetic effect is included in the model.

$$\left[\begin{array}{c}{\mathbf{a}}_{\mathbf{F}}\\ {\mathbf{a}}_{\mathbf{M}}\\ \begin{array}{c}{\mathbf{m}}_{\mathbf{F}}\\ {\mathbf{m}}_{\mathbf{M}}\end{array}\end{array}\right]\sim MVN\left[0, \mathbf{A} \otimes \left(\begin{array}{c}\begin{array}{c}{\sigma }_{{a}_{F}}^{2}\\ {\sigma }_{{a}_{F}{a}_{M}}\end{array}\\ \begin{array}{c}0\\ 0\end{array}\end{array} \begin{array}{c}\begin{array}{c}{\sigma }_{{a}_{F}{a}_{M}}\\ {\sigma }_{{a}_{M}}^{2}\end{array}\\ \begin{array}{c}0\\ 0\end{array}\end{array} \begin{array}{c}\begin{array}{c}0\\ 0\end{array}\\ \begin{array}{c}{\sigma }_{{m}_{F}}^{2}\\ {\sigma }_{{m}_{F}{m}_{M}}\end{array}\end{array} \begin{array}{c}\begin{array}{c}0\\ 0\end{array}\\ \begin{array}{c}{\sigma }_{{m}_{F}{m}_{M}}\\ {\sigma }_{{m}_{M}}^{2}\end{array}\end{array}\right)\right],$$

$$\left[\begin{array}{c}{\mathbf{e}}_{\mathbf{F}}\\ {\mathbf{e}}_{\mathbf{M}}\\ \begin{array}{c}{\mathbf{p}\mathbf{e}}_{\mathbf{F}}\\ {\mathbf{p}\mathbf{e}}_{\mathbf{M}}\end{array}\end{array}\right]\sim MVN\left[0, \mathbf{I} \otimes \left(\begin{array}{c}\begin{array}{c}{\sigma }_{{e}_{F}}^{2}\\ 0\end{array}\\ \begin{array}{c}0\\ 0\end{array}\end{array} \begin{array}{c}\begin{array}{c}0\\ {\sigma }_{{e}_{M}}^{2}\end{array}\\ \begin{array}{c}0\\ 0\end{array}\end{array} \begin{array}{c}\begin{array}{c}0\\ 0\end{array}\\ \begin{array}{c}{\sigma }_{{pe}_{F}}^{2}\\ {\sigma }_{{pe}_{F}{pe}_{M}}\end{array}\end{array} \begin{array}{c}\begin{array}{c}0\\ 0\end{array}\\ \begin{array}{c}{\sigma }_{{pe}_{F}{pe}_{M}}\\ {\sigma }_{p{e}_{M}}^{2}\end{array}\end{array}\right)\right].$$

The third model, the “Coram model,” assumes that the genetic covariance is different from 0 and is the commonly used model to study direct and maternal effects in many species [2, 3].

$$\left[\begin{array}{c}{\mathbf{a}}_{\mathbf{F}}\\ {\mathbf{a}}_{\mathbf{M}}\\ \begin{array}{c}{\mathbf{m}}_{\mathbf{F}}\\ {\mathbf{m}}_{\mathbf{M}}\end{array}\end{array}\right]\sim MVN\left[0, \mathbf{A} \otimes \left(\begin{array}{c}\begin{array}{c}{\sigma }_{{a}_{F}}^{2}\\ {\sigma }_{{a}_{F}{a}_{M}}\end{array}\\ \begin{array}{c}{\sigma }_{{a}_{F}{m}_{F}}\\ {\sigma }_{{a}_{F}{m}_{M}}\end{array}\end{array} \begin{array}{c}\begin{array}{c}{\sigma }_{{a}_{F}{a}_{M}}\\ {\sigma }_{{a}_{M}}^{2}\end{array}\\ \begin{array}{c}{\sigma }_{{a}_{M}{m}_{F}}\\ {\sigma }_{{a}_{M}{m}_{M}}\end{array}\end{array} \begin{array}{c}\begin{array}{c}{\sigma }_{{a}_{F}{m}_{F}}\\ {\sigma }_{{a}_{M}{m}_{F}}\end{array}\\ \begin{array}{c}{\sigma }_{{m}_{F}}^{2}\\ {\sigma }_{{m}_{F}{m}_{M}}\end{array}\end{array} \begin{array}{c}\begin{array}{c}{\sigma }_{{a}_{F}{m}_{M}}\\ {\sigma }_{{a}_{M}{m}_{M}}\end{array}\\ \begin{array}{c}{\sigma }_{{m}_{F}{m}_{M}}\\ {\sigma }_{{m}_{M}}^{2}\end{array}\end{array}\right)\right],$$

$$\left[\begin{array}{c}{\mathbf{e}}_{\mathbf{F}}\\ {\mathbf{e}}_{\mathbf{M}}\\ \begin{array}{c}{\mathbf{p}\mathbf{e}}_{\mathbf{F}}\\ {\mathbf{p}\mathbf{e}}_{\mathbf{M}}\end{array}\end{array}\right]\sim MVN\left[0, \mathbf{I} \otimes \left(\begin{array}{c}\begin{array}{c}{\sigma }_{{e}_{F}}^{2}\\ 0\end{array}\\ \begin{array}{c}0\\ 0\end{array}\end{array} \begin{array}{c}\begin{array}{c}0\\ {\sigma }_{{e}_{M}}^{2}\end{array}\\ \begin{array}{c}0\\ 0\end{array}\end{array} \begin{array}{c}\begin{array}{c}0\\ 0\end{array}\\ \begin{array}{c}{\sigma }_{{pe}_{F}}^{2}\\ {\sigma }_{{pe}_{F}{pe}_{M}}\end{array}\end{array} \begin{array}{c}\begin{array}{c}0\\ 0\end{array}\\ \begin{array}{c}{\sigma }_{{pe}_{F}{pe}_{M}}\\ {\sigma }_{p{e}_{M}}^{2}\end{array}\end{array}\right)\right].$$

The fourth model, the “Corepe model,” assumes that the environmental covariance between dam BW and the permanent effects of the dam on offspring is different from zero and that the genetic covariance between maternal and direct genetic effects is zero.

$$\left[\begin{array}{c}{\mathbf{a}}_{\mathbf{F}}\\ {\mathbf{a}}_{\mathbf{M}}\\ \begin{array}{c}{\mathbf{m}}_{\mathbf{F}}\\ {\mathbf{m}}_{\mathbf{M}}\end{array}\end{array}\right]\sim MVN\left[0, \mathbf{A} \otimes \left(\begin{array}{c}\begin{array}{c}{\sigma }_{{a}_{F}}^{2}\\ {\sigma }_{{a}_{F}{a}_{M}}\end{array}\\ \begin{array}{c}0\\ 0\end{array}\end{array} \begin{array}{c}\begin{array}{c}{\sigma }_{{a}_{F}{a}_{M}}\\ {\sigma }_{{a}_{M}}^{2}\end{array}\\ \begin{array}{c}0\\ 0\end{array}\end{array} \begin{array}{c}\begin{array}{c}0\\ 0\end{array}\\ \begin{array}{c}{\sigma }_{{m}_{F}}^{2}\\ {\sigma }_{{m}_{F}{m}_{M}}\end{array}\end{array} \begin{array}{c}\begin{array}{c}0\\ 0\end{array}\\ \begin{array}{c}{\sigma }_{{m}_{F}{m}_{M}}\\ {\sigma }_{{m}_{M}}^{2}\end{array}\end{array}\right)\right],$$

$$\left[\begin{array}{c}{\mathbf{e}}_{\mathbf{F}}\\ {\mathbf{e}}_{\mathbf{M}}\\ \begin{array}{c}{\mathbf{p}\mathbf{e}}_{\mathbf{F}}\\ {\mathbf{p}\mathbf{e}}_{\mathbf{M}}\end{array}\end{array}\right]\sim MVN\left[0, \mathbf{I} \otimes \left(\begin{array}{c}\begin{array}{c}{\sigma }_{{e}_{F}}^{2}\\ 0\end{array}\\ \begin{array}{c}{\sigma }_{{e}_{F}{pe}_{F}}\\ {\sigma }_{{e}_{F}{pe}_{M}}\end{array}\end{array} \begin{array}{c}\begin{array}{c}0\\ {\sigma }_{{e}_{M}}^{2}\end{array}\\ \begin{array}{c}0\\ 0\end{array}\end{array} \begin{array}{c}\begin{array}{c}{\sigma }_{{e}_{F}{pe}_{F}}\\ 0\end{array}\\ \begin{array}{c}{\sigma }_{{pe}_{F}}^{2}\\ {\sigma }_{{pe}_{F}{pe}_{M}}\end{array}\end{array} \begin{array}{c}\begin{array}{c}{\sigma }_{{e}_{F}{pe}_{M}}\\ 0\end{array}\\ \begin{array}{c}{\sigma }_{{pe}_{F}{pe}_{M}}\\ {\sigma }_{p{e}_{M}}^{2}\end{array}\end{array}\right)\right].$$

Note that an environmental covariance exists between the residual of the dam and her subsequent maternal environmental effect on both females and males (${\sigma }_{{e}_{F}{pe}_{F}}$ and${\sigma }_{{e}_{F}{pe}_{M}}$). However, it is not possible to estimate a covariance between the residual of males and the environmental maternal effect ${(\sigma }_{{e}_{M}{pe}_{M}}$ or ${\sigma }_{{e}_{M}{pe}_{F} })$ because the male parents do not contribute to maternal effects. For this reason, the corresponding covariance was set to 0. In our study, the maternal environmental effect (pe) was computed using full-sibs within each sex group. Therefore, the dam had two pe: one on its female offspring and one on its male offspring, and the covariance between them was estimated. This means that two direct-maternal environmental covariances were estimated.

Finally the most complex model, the “Coramepe model” assumes that both the genetic and environmental covariances are different from 0.

$$\left[\begin{array}{c}{\mathbf{a}}_{\mathbf{F}}\\ {\mathbf{a}}_{\mathbf{M}}\\ \begin{array}{c}{\mathbf{m}}_{\mathbf{F}}\\ {\mathbf{m}}_{\mathbf{M}}\end{array}\end{array}\right]\sim MVN\left[0, \mathbf{A}\otimes \left(\begin{array}{c}\begin{array}{c}{\sigma }_{{a}_{F}}^{2}\\ {\sigma }_{{a}_{F}{a}_{M}}\end{array}\\ \begin{array}{c}{\sigma }_{{a}_{F}{m}_{F}}\\ {\sigma }_{{a}_{F}{m}_{M}}\end{array}\end{array} \begin{array}{c}\begin{array}{c}{\sigma }_{{a}_{F}{a}_{M}}\\ {\sigma }_{{a}_{M}}^{2}\end{array}\\ \begin{array}{c}{\sigma }_{{a}_{M}{m}_{F}}\\ {\sigma }_{{a}_{M}{m}_{M}}\end{array}\end{array} \begin{array}{c}\begin{array}{c}{\sigma }_{{a}_{F}{m}_{F}}\\ {\sigma }_{{a}_{M}{m}_{F}}\end{array}\\ \begin{array}{c}{\sigma }_{{m}_{F}}^{2}\\ {\sigma }_{{m}_{F}{m}_{M}}\end{array}\end{array} \begin{array}{c}\begin{array}{c}{\sigma }_{{a}_{F}{m}_{M}}\\ {\sigma }_{{a}_{M}{m}_{M}}\end{array}\\ \begin{array}{c}{\sigma }_{{m}_{F}{m}_{M}}\\ {\sigma }_{{m}_{M}}^{2}\end{array}\end{array}\right)\right],$$

$$\left[\begin{array}{c}{\mathbf{e}}_{\mathbf{F}}\\ {\mathbf{e}}_{\mathbf{M}}\\ \begin{array}{c}{\mathbf{p}\mathbf{e}}_{\mathbf{F}}\\ {\mathbf{p}\mathbf{e}}_{\mathbf{M}}\end{array}\end{array}\right]\sim MVN\left[0, \mathbf{I}\otimes \left(\begin{array}{c}\begin{array}{c}{\sigma }_{{e}_{F}}^{2}\\ 0\end{array}\\ \begin{array}{c}{\sigma }_{{e}_{F}{pe}_{F}}\\ {\sigma }_{{e}_{F}{pe}_{M}}\end{array}\end{array} \begin{array}{c}\begin{array}{c}0\\ {\sigma }_{{e}_{M}}^{2}\end{array}\\ \begin{array}{c}0\\ 0\end{array}\end{array} \begin{array}{c}\begin{array}{c}{\sigma }_{{e}_{F}{pe}_{F}}\\ 0\end{array}\\ \begin{array}{c}{\sigma }_{{pe}_{F}}^{2}\\ {\sigma }_{{pe}_{F}{pe}_{M}}\end{array}\end{array} \begin{array}{c}\begin{array}{c}{\sigma }_{{e}_{F}{pe}_{M}}\\ 0\end{array}\\ \begin{array}{c}{\sigma }_{{pe}_{F}{pe}_{M}}\\ {\sigma }_{p{e}_{M}}^{2}\end{array}\end{array}\right)\right].$$

All statistical analyses were performed using the DMU software package [18].

### Simulated populations

Using the stochastic simulation program ADAM [19], we simulated an idealized breeding population that mimics the breeding program of real data. The simulated data included 40 SR, with offspring being hatched and phenotyped in each SR, and selection being applied after genetic evaluation using an animal model, see Chu et al. [20] for the descriptions of SR and overlapping SR in broiler breeding. Sex was randomly assigned to the offspring with a 1:1 ratio. In each SR, 7800 birds were simulated with 52 sires and 520 dams. After measuring BW, the birds were evaluated using a pedigree-based BLUP model or ranked at random. In each SR, 13 males and 130 females were selected, therefore one male was mated with 10 females. These birds were later used as sires and dams to create the next generation. For SR 1 to 6, the selection of the parents was random. The main purpose of this step was to build up data for the estimation of variance components, which was done after SR 6. From rounds 7 to 40, selection of the parents was based on estimated breeding values (EBV) obtained from pedigree-based BLUP that used the variance components estimated after SR 6. For the estimation of both variance components and breeding values, we used a univariate version of the Moda model. Across the 40 SR, a dataset of 312,000 birds was accumulated. The number of offspring per dam and per hatch decreased as its age increased ranging from 2.49 offspring per dam at early age to 2.15 offspring at later age.

The phenotypes of individuals were simulated using the infinitesimal genetic model, based on the following model:

$$\mathbf{B}\mathbf{W}=\mathbf{X}\mathbf{b}+\mathbf{Z}\mathbf{a}+\mathbf{W}\mathbf{m}+\mathbf{W}\mathbf{p}\mathbf{e}+\mathbf{B}\mathbf{e},$$

where $\mathbf{X}$, $\mathbf{Z}$, $\mathbf{W}$ and $\mathbf{B}$ are the incidence matrices for the fixed effect, the additive genetic effect, the maternal effect and the environmental effect, respectively. $\mathbf{b}$, $\mathbf{a}$, $\mathbf{m}$, $\mathbf{p}\mathbf{e}$ and $\mathbf{e}$ are the vectors of the fixed effects (hatch within each SR, sex and the dam age in weekly classes), the additive genetic effect, the maternal genetic effect, the permanent environmental maternal effect and the residual environmental effect, respectively. The (co)variance components used to simulate the population assumed non-null direct-maternal genetic and environmental covariances and thus, was a univariate version of the Coramepe model. The (co)variance components used in the simulation were those estimated with the real data as shown in Table 5. The simulations were done 15 times.

### Cross-validation

To assess the predictive ability of our evaluations, we focused on the accuracy of the prediction of breeding values predicted by each model and on the inflation of those breeding values, and we used different CV strategies (forward prediction and half-sib prediction) and predictors, which are described below. Variance components were always estimated using the full dataset (training and validation population together). Before computing the correlations and inflations, all the estimated breeding values were corrected for genetic trend. This was done using a multivariate analysis of the variance approach. Thus, the variances and covariances conditional on the genetic trend were used to estimate the correlation, and inflation was corrected for the effect of SR.

#### Forward prediction

We divided our data into two subsets: a training dataset that consisted of the first SR and a validation dataset that included the population of the last five SR. The breeding values of the individuals of the validation population were predicted using information on past relatives only, and thus their own phenotypes were masked (denoted as reduced dataset), and using all the phenotypes, thus their own performance was included (denoted as full dataset). In the first case, we will refer to the breeding values as EBV_reduced_ and in the second case, as EBV_full_.

The accuracy of prediction for each model was computed as the correlation between the breeding values of the individuals estimated without their phenotypes (EBV_reduced_) and (1) the phenotype corrected for the fixed effect (*y*_c_) and (2) the breeding values estimated including their phenotypes (EBV_full_). In the following sections, these correlations will be referred to as “cor(*y*_c_, EBV_reduced_)” and “cor(EBV_full_, EBV_reduced_),” respectively. The “cor(EBV_full_, EBV_reduced_)” corresponds to the method proposed by Legarra and Reverter [12].

Inflation of predicted breeding values is an important component when comparing models for genetic evaluation because it affects the comparison of birds that are affected differently by inflation. Here, the inflation of breeding values, sometimes incorrectly called bias, was estimated as the slope of the regression of the response on the predictor. When the breeding values are neither inflated nor deflated, the expected value of this slope is 1. The predictor used to estimate inflation was in all cases the EBV_reduced_. However, two different responses were used: (1) the phenotype corrected for fixed effect (*y*_c_), which will be referred to as “*y*_c_ ~ EBV_reduced_” and (2) the breeding values estimated using the full datasets (EBV_full_) as proposed by Legarra and Reverter [12], which will be referred to as EBV_full_ ~ EBV_reduced_.

#### Half-sibs prediction

This strategy follows the proposal of Chu et al. [13] to assess the predictive ability of the model in the presence of maternal effects. In this case, the paternal half-sib groups in the last five SR were randomly divided into two groups. Half of the individuals were kept in the training population and the other half were assigned to the validation population. Therefore, the breeding values of the individuals from the validation population are estimated using information from their ancestors, as before, but also from 50% of their paternal half-sib.

Within the validation population, paternal half-sibs were randomly paired. For the real data, because performances in males and females were considered as different traits, the pairing of the half-sibs was done within each sex. Thus, 5267 pairs of paternal half-sisters and 5147 pairs of paternal half-brothers were created, which were the maximal number of pairs possible. The sampling of pairs was performed 50 times. Here, the predictive ability of the model was defined as the correlation between the phenotype corrected for the fixed effects of an individual $i$ with the EBV_reducedHS_ of its paternal half-sib $j$. In the following sections, we will refer to this correlation as “Halfsibs cor(*y*_c_, EBV_reducedHS_)”. Compared with the cor(*y*_c_, EBV_reduced_), described in the “Forward prediction” section, where *y*_c_ and EBV_reduced_ belonged to the same individuals, here the *y*_c_ belonged to paired paternal half-sibs.

The same tests as described above were used to assess the predictive ability of the models in the simulated populations. However, in this case, the true breeding values (TBV) were known. Therefore, to assess the accuracy of the prediction of the models, the correlation between the breeding values of the individuals estimated without their phenotypes and the TBV (cor(TBV, EBV_reduced_)) was also computed, and this is considered to be the most accurate test. In the same way, a third test was added for the inflation of the breeding value. This test corresponds to the slope of the regression of the breeding values of the individuals estimated without their phenotypes on the TBV, which will be referred to as TBV ~ EBV_reduced_. In the simulation, BW was modeled as being the same trait in males and females. Therefore, the paternal half-sibs pairs were done across sex resulting in 10,855 pairs. Moreover, for the simulation study, the reported accuracy of prediction and inflation of breeding values are the average of 15 replicates.

## Results

### Summary of the data

The numbers of males and females phenotyped for BW were similar. Males were 11% heavier than females, i.e. 2066 g vs. 1859 g as presented in Table 1, which also shows that BW was more variable for males than for females with the standard deviation (SD) for males being 29% larger than for females.

### Direct-maternal genetic and environmental covariance in real data

#### Model fit

All the models used were compared with the Coramepe model based on log-likelihood (see Table 2). The Coramepe model provides the best fit to the data. Every time a component was added to the model, the fit of the model improved significantly compared with the previous model (Table 2).

**Table 2:** (Co)-variances, correlations, and likelihood test estimated using the real data per model and sex

	Moda		Modam		Coram		Corepe		Coramepe	
	f	m	f	m	f	m	f	m	f	m
${{{\sigma}}}_{{{a}}}^{2}$	5967 *(267)*	9093 *(446)*	5436 *(269)*	8179 *(442)*	6090 *(370)*	9559 *(624)*	4469 *(282)*	7065*(447)*	4952 *(340)*	8170 *(568)*
${{{\sigma}}}_{{{a}}{{m}}}$	–	–	–	–	–555*(162)*	–1187*(273)*	–	–	–795*(178)*	–1436*(292)*
${{{\sigma}}}_{{{m}}}^{2}$	–	–	550 *(86)*	818 *(133)*	698 *(109)*	121 *(193)*	657 *(94)*	956 *(144)*	1016 *(146)*	1608 *(237)*
${{{\sigma}}}_{{{e}}}^{2}$	14,259 *(143)*	23,065 *(241)*	14,520 *(144)*	23,519 *(239)*	14,196 *(192)*	22,833 *(324)*	15,011 *(152)*	24,072 *(241)*	14,772 *(179)*	23,523 *(297)*
${{{\sigma}}}_{{{e}}{{p}}{{e}}}$	–	–	–	–	–	–	871*(118)*	1055*(148)*	1048*(130)*	1232*(164)*
${{{\sigma}}}_{{{p}}{{e}}}^{2}$	792 *(47)*	1380 *(81)*	473 *(57)*	931 *(96)*	553 *(61)*	1062 *(99)*	619 *(64)*	1103 *(103)*	727 *(68)*	1254 *(107)*
${{{r}}}_{{{a}}{{m}}}$	–	–	–	–	–0.27 *(0.06)*	–0.35 *(0.06)*	–	–	–0.35 *(0.06)*	–0.40 *(0.06)*
${{{r}}}_{{{e}}{{p}}{{e}}}$	–	–	–	–	–	–	0.29 *(0.03)*	0.26 *(0.03)*	0.32 *(0.04)*	0.29 *(0.04)*
${{{h}}}^{2}$	0.28*(0.01)*	0.27*(0.01)*	0.26*(0.01)*	0.24*(0.01)*	0.28 *(0.01)*	0.28 *(0.02)*	0.22 *(0.01)*	0.21 *(0.01)*	0.23 *(0.01)*	0.24 *(0.01)*
LRT	214		81		55		36		0	

Additive genetic variance ($${\sigma }_{a}^{2}$$), maternal genetic variance ($${\sigma }_{m}^{2}$$), environmental variance ($${\sigma }_{e}^{2}$$), environmental permanent maternal variance ($${\sigma }_{pe}^{2}$$), genetic, environmental covariances and correlations ($${\sigma }_{am}$$, $${\sigma }_{epe}$$, $${r}_{am}$$, $${r}_{epe}$$), heritability ($${h}^{2})$$ and likelihood ratio test (LRT) estimated with the real data for the five models relative to the Coramepe model

Moda is the model for which no maternal genetic effects are included and for which the direct-maternal environmental covariance is null. Modam is the model for which both the direct-maternal genetic and the direct-maternal environmental covariance are null. Coram is the model for which the direct-maternal genetic effect is considered as non-null while the direct-maternal environmental covariance is null. Corepe is the model for which the direct-maternal environmental effect is considered as non-null while the direct-maternal genetic covariance is null. Coramepe is the model for which both the direct-maternal genetic and the direct-maternal environmental covariance are non-null

Standard errors of the estimates are in italics between brackets

#### (Co)variance components

The (co)variance components estimated using the real dataset for each of the five models are presented in Table 2. The genetic correlations between the additive genetic effect and the genetic maternal effect were negative (from –0.27 to –0.40), while the correlation between the residual environmental effect of the dam and the permanent environmental maternal effect on the offspring of the dam were positive (from 0.26 to 0.32).

Adding genetic (the Coram model) or environmental (the Corepe model) covariance to the model led to an increase in the estimates of both the genetic and environmental maternal effects. This increase was greater when both covariance components were added to the model (the Coramepe model).

Adding environmental covariance led to a decrease in the estimated additive genetic variance and an increase in the estimated environmental variance, which is reflected by a decrease in the estimate of the heritability (from 0.28 to 0.22 and from 0.28 to 0.21, in females and males, respectively). This decrease in the estimate of the heritability was even greater when the genetic covariance was added to the model. In contrast, adding the genetic covariance only led to an increase in the estimate of the additive genetic variance, which is reflected by an increase in the estimate of the heritability.

It is interesting to note that ignoring the direct-maternal environmental covariance seems to lead to an estimate of direct-maternal covariance that is less negative. These findings highlight the changes in the definition of the direct additive genetic variance using different models. If the direct-maternal genetic or environmental covariances are non-null, then ignoring them may induce bias in the estimation of variance components and in the prediction of breeding values.

#### Predictive ability and inflation of breeding values

The observed accuracies of predicted breeding values in the real data using three different tests are in Table 3. The first two tests, cor(*y*_c_, EBV_reduced_) and Halfsibs cor(*y*_c_, EBV_reducedHS_), relied on the correlation between the breeding values estimated for the individuals of the validation population using the training dataset (EBV_reduced_) and the phenotypes corrected for the fixed effects (*y*_c_). The third test, cor(EBV_full_, EBV_reduced_) is the correlation between the breeding values estimated with the full dataset (EBV_full_) and the breeding value estimated using the reduced dataset (EBV_reduced_).

**Table 3:** Observed accuracies of prediction estimated for the five models per sex

	Moda		Modam		Coram		Corepe		Coramepe	
	f	m	f	m	f	m	f	m	f	m
cor(*y*_c_, EBV_reduced_)	0.216	0.248	0.205	0.241	0.202	0.239	0.199	0.235	0.192	0.230
Halfsibs cor(*y*_c_, EBV_reducedHS_)	0.197	0.203	0.198	0.205	0.197	0.205	0.196	0.203	0.195	0.202
cor(EBV_full_, EBV_reduced_)	0.614	0.679	0.595	0.669	0.587	0.659	0.618	0.686	0.614	0.680

Observed accuracies of prediction are defined as the correlation between the phenotype corrected for fixed effects (*y*_c_) and the predicted breeding values (EBV_reduced_), either when these values were estimated for the same individual (cor(*y*_c_, EBV_reduced_)) or for paternal half-sibs pairs (Halfsibs cor(*y*_c_, EBV_reducedHS_)). The third definition of the accuracy of prediction cor(EBV_full_, EBV_reduced_) is defined as the correlation between the breeding values estimated with the full dataset (EBV_full_) and the one estimated using the reduced dataset (EBV_reduced_). Moda is the model for which no maternal genetic effects are included and for which the direct-maternal environmental covariance is null. Modam is the model for which both the direct-maternal genetic and the direct-maternal environmental covariance are null. Coram is the model for which the direct-maternal genetic effect is considered as non-null while the direct-maternal environmental covariance is null. Corepe is the model for which the direct-maternal environmental effect is considered as non-null while the direct-maternal genetic covariance is null. Coramepe is the model for which both the direct-maternal genetic and the direct-maternal environmental covariance are non-null

The observed accuracies for each model, defined as cor(*y*_c_, EBV_reduced_), ranged from 0.192 to 0.216 in females and from 0.230 to 0.248 in males (Table 3). For this first definition of accuracy, the Moda model that does not include a maternal genetic effect gave the highest observed accuracy.

An alternative measure of accuracy, defined as Halfsibs cor(*y*_c_, EBV_reducedHS_), ranged from 0.195 to 0.197 in females and from 0.202 to 0.205 in males (Table 3). For this second definition of accuracy, the Modam model tended to give the highest observed accuracy.

The Legarra–Reverter correlation, defined as cor(EBV_full_, EBV_reduced_), ranged from 0.587 to 0.618 in females and from 0.659 to 0.680 in males (Table 3). For this last definition of accuracy, the Corepe model tended to show the highest accuracy for both sexes. Overall observed accuracies were higher in males than in females.

Inflations of the breeding values, for the real dataset, obtained by using two different responses, *y*_c_ and EBV_full_, are presented in Table 4. When considering the corrected phenotype as the response, the inflation of breeding values ranged from 1.131 to 1.235 for females and from 1.145 to 1.266 for males. For both sexes, Moda was the model that resulted in the least deflated breeding values and Coram was the model that resulted in the most deflated breeding values. When the breeding values estimated using the full dataset are considered as the response, the inflation of the breeding value ranged from 1.010 to 1.049 for females and from 1.048 to 1.077 for males. Here, Coramepe was the model that resulted in the most deflated breeding values.

**Table 4:** Inflation of the estimated breeding values for the five models per sex

	Moda		Modam		Coram		Corepe		Coramepe	
	f	m	f	m	f	m	f	m	f	m
*y*_c_ ~ EBV_reduced_	1.131	1.145	1.181	1.240	1.235	1.266	1.153	1.206	1.207	1.235
EBV_full_ ~ EBV_reduced_	1.033	1.048	1.010	1.050	1.019	1.058	1.035	1.065	1.049	1.077

The inflations were estimated for each model. The first one is the slope of the regression of the phenotype corrected for fixed effects (*y*_c_) on the predicted breeding values (EBV_reduced_). The second one is the slope of the regression of predicted breeding values using the full dataset (EBV_full_) on the predicted breeding values using the reduced dataset (EBV_reduced_). Moda is the model for which no maternal genetic effects are included and for which the direct-maternal environmental covariance is null. Modam is the model for which both the direct-maternal genetic and the direct-maternal environmental covariance are null. Coram is the model for which the direct-maternal genetic effect is considered as non-null while the direct-maternal environmental covariance is null. Corepe is the model for which the direct-maternal environmental effect is considered as non-null while the direct-maternal genetic covariance is null. Coramepe is the model for which both the direct-maternal genetic and the direct-maternal environmental covariance are non-null

To summarize, depending on the test used, the models rank differently. If we focus only on the accuracy of prediction of the models, the Moda model (not including maternal genetic effect and no direct-maternal covariance) will be the best model if the test used is cor(*y*_c_, EBV_reduced_). However, if the cor(EBV_full_, EBV_reduced_) is used, then the Corepe model (including both genetic and environmental effects but only the direct-maternal environmental covariance) yields the best accuracy. Finally, if the test used is Halfsibs cor(*y*_c_, EBV_reducedHS_), then the Modam model (including both genetic and environmental maternal effect but no direct-maternal covariance) results in the highest predictive ability. Regarding the inflation of the breeding values, when the corrected phenotype was considered as the response, the best model was Moda, which does not include a maternal genetic effect and no direct-maternal covariance. In contrast, when the breeding values estimated using the full dataset are considered as the response, the Coramepe model (including both direct-maternal covariances) was the best model. Traditionally, the predictive ability of the models is estimated using cor(*y*_c_, EBV_reduced_) or cor(EBV_full_, EBV_reduced_), but in the case of a trait influenced by maternal effects, it seems that the conclusions will differ depending on the tests used. In terms of inflation of breeding values, it will even lead to opposite conclusions. Because of the conflicting results, a simulation study was conducted to understand why the different tests led to such different rankings of the models. The results of the simulation study are presented in the following section.

### Direct-maternal genetic and environmental covariances in the simulated populations

#### Model fit

Based on the likelihood, Coramepe, which is the true model, is, not surprisingly, the model that gives the best fit to the data. Every time a component was added to the model, its fit improved significantly (Table 5). The ranking of the models and the difference in log(L) are similar with simulated and real data. This suggests that the simulation model generates the same complexity as the real data.

**Table 5:** (Co)-variances, correlations and likelihood test estimated with the simulated data per model

	Moda	Modam	Coram	Corepe	Coramepe	True VC
${{{\sigma}}}_{{{a}}}^{2}$	9217 *(400)*	8680 *(358)*	10,251 *(601)*	6997 *(292)*	7937 *(332)*	8046
${{{\sigma}}}_{{{m}}}^{2}$	–	430 *(96)*	609 *(125)*	583 *(118)*	864 *(193)*	829
${{{\sigma}}}_{{{p}}{{e}}}^{2}$	743 *(50)*	515 *(72)*	664 *(76)*	737 *(74)*	879 *(82)*	906
${{{\sigma}}}_{{{e}}}^{2}$	19,302 *(211)*	19,554 *(188)*	18,810 *(290)*	20,364 *(157)*	19,918 *(153)*	19,860
${{{\sigma}}}_{{{a}}{{m}}}$	–	–	–986 *(186)*	–	–919 *(205)*	–912
${{{\sigma}}}_{{{e}}{{p}}{{e}}}$	–	–	–	1223 *(126)*	1291 *(146)*	1272
LRT	163	70	52	16	0	NA

Additive genetic variance (${\sigma }_{a}^{2}$), maternal genetic variance (${\sigma }_{m}^{2}$), environmental permanent maternal variance (${\sigma }_{pe}^{2}$), environmental variance (${\sigma }_{e}^{2}$), genetic, environmental covariances (${\sigma }_{am}$, ${\sigma }_{epe}$) estimated in the simulated data for the five models and the one used to simulate the population (True VC) and the likelihood ratio test (LRT). Standard deviations of the estimates among the 15 replicates are shown in italics between brackets. Moda is the model for which no maternal genetic effects are included and for which the direct-maternal environmental covariance is null. Modam is the model for which both the direct-maternal genetic and the direct-maternal environmental covariance are null. Coram is the model for which the direct-maternal genetic effect is considered as non-null while the direct-maternal environmental covariance is null. Corepe is the model for which the direct-maternal environmental effect is considered as non-null while the direct-maternal genetic covariance is null. Coramepe is the model for which both the direct-maternal genetic and the direct-maternal environmental covariance are non-null

#### (Co)variance components

In the simulation, the (co)variance components estimated with the true prediction model, denoted as the Coramepe model, on the full dataset, after 40 SR were similar to the one used to simulate the population (Table 5).

With the Moda model, which did not include a maternal genetic effect, the additive genetic variance was overestimated compared with the true direct additive genetic variance. In fact, the additive genetic variance in this case was almost equal to the sum of the true direct additive genetic variance and the true maternal genetic variance.

When ignoring both direct-maternal genetic and environmental covariances (Modam model), the maternal genetic variance and the permanent environmental maternal were halved compared with the true variance components.

When adding the direct-maternal genetic covariance (Coram model), the direct additive genetic variance was highly overestimated, whereas the residual variance was underestimated compared with the true variance components.

When adding the direct-maternal environmental covariance (Corepe model), the additive genetic variance was highly underestimated, whereas the residual variance was overestimated compared with the true variance components.

#### Accuracy and inflation of predicted breeding values

Accuracies of prediction in the simulated dataset using four different tests are in Table 6. In the first test, the breeding value estimated with the reduced dataset (EBV_reduced_) of an animal from the validation population was correlated with the TBV (referred to as cor(TBV, EBV_reduced_)). In the second test, the EBV_reduced_ of birds from the validation population was correlated with the phenotype corrected for the fixed effect (referred to as cor(*y*_c_, EBV_reduced_)). In the third test, the EBV_reduced_ of one animal from the validation population was correlated with the phenotype corrected for the fixed effect of one of its paternal half-sibs from the validation population (referred to as Halfsibs cor(*y*_c_, EBV_reducedHS_)). Finally, in the last test, the EBV_reduced_ of birds from the validation population estimated without including their phenotype was correlated with the EBV_full_ estimated using their own phenotype (referred to as cor(EBV_full_, EBV_reduced_)). The four different correlations cannot be compared directly, however, the ranking of the models can.

**Table 6:** Observed accuracies of prediction estimated per model on the simulated dataset using four tests

	Moda	Modam	Coram	Corepe	Coramepe	se
cor(TBV, EBV_reduced_)	0.352	0.369	0.369	0.373	0.374	0.010
cor(*y*_c_, EBV_reduced_)	0.162	0.160	0.158	0.157	0.154	0.004
Halfsibs cor(*y*_c_, EBV_reducedHS_)	0.203	0.205	0.206	0.209	0.211	0.005
cor(EBV_full_, EBV_reduced_)	0.596	0.609	0.604	0.627	0.628	0.010

Observed accuracies of prediction are defined as the correlation between the true breeding values and the predicted breeding values (cor(TBV, EBV_reduced_)); the phenotype corrected for fixed effects (*y*_c_) and the predicted breeding values (EBV_reduced_), either when these values were estimated for the same individual (cor(*y*_c_, EBV_reduced_)) or for paternal half-sibs pairs (Halfsibs cor(*y*_c_, EBV_reducedHS_)) and the breeding values estimated with the full dataset (EBV_full_) and the one estimated using the reduced dataset (EBV_reduced_). The accuracies were estimated for each model. The standard errors (se) are presented in the last column. Moda is the model for which no maternal genetic effects are included and for which the direct-maternal environmental covariance is null. Modam is the model for which both the direct-maternal genetic and the direct-maternal environmental covariance are null. Coram is the model for which the direct-maternal genetic effect is considered as non-null while the direct-maternal environmental covariance is null. Corepe is the model for which the direct-maternal environmental effect is considered as non-null while the direct-maternal genetic covariance is null. Coramepe is the model for which both the direct-maternal genetic and the direct-maternal environmental covariance are non-null

With the first method, cor(TBV, EBV_reduced_), accuracies of prediction ranged from 0.352 to 0.374 (Table 6). A gain in accuracy of 4.69% (standard error (se) = 1.16) compared to the Moda model, which did not include a maternal genetic effect, was observed when the maternal genetic effect was added to the model. This gain increased up to 6.26% (se = 1.47) when both direct-maternal covariances were added to the prediction model (the Coramepe model).

With the second method, cor(*y*_c_, EBV_reduced_), accuracies of prediction ranged from 0.154 to 0.162, with the Coramepe model resulting in the lowest accuracy and the standard model resulting in the highest accuracy (Table 6). However, compared to the Moda model, an apparent loss in accuracy was observed when the maternal genetic effect was added to the model, which was largest when genetic and/or environmental covariance were added to the prediction model.

With the third method, Halfsibs cor(*y*_c_, EBV_reducedHS_), accuracies of prediction ranged from 0.203 to 0.211. The Coramepe model resulted in the highest accuracy and the Moda model in the lowest accuracy (Table 6). A gain in accuracy of 0.67% (se = 0.46) compared to the Moda model, which did not include a maternal genetic effect, was observed when the maternal genetic effect was added to the model. This gain increased up to 3.35% (se = 0.79) when both genetic and environmental direct-maternal covariances were added to the prediction model.

With the last method, cor(EBV_full_, EBV_reduced_), the accuracies of prediction ranged from 0.596 to 0.628. Here again, the Coramepe model resulted in the highest accuracy and the standard model in the lowest accuracy (Table 6). A gain in accuracy of 1.93% (se = 0.49) compared to the Moda model, which did not include a maternal genetic effect, was observed when the maternal genetic effect was added to the model. This gain increased up to 5.42% (se = 0.79) when both direct-maternal covariances were added to the prediction model.

Overall, the ranking of the models using cor(TBV, EBV_reduced_), which is the most accurate test, Halfsibs cor(*y*_c_, EBV_reducedHS_) and cor(EBV_full_, EBV_reduced_) were identical. However using the cor(*y*_c_, EBV_reduced_) as is commonly done in animal breeding leads to a biased ranking of models in terms of prediction accuracy.

Inflation of the breeding values, for the simulated dataset, using three different estimates is shown in Table 7. When considering the TBV as a response, the inflation of the breeding values ranged from 0.844 to 1.025. When the corrected phenotype is considered as the response, the inflation of the breeding values ranged from 0.967 to 0.998. Finally, when the breeding value estimated using the full dataset is considered as the response, the inflation of the breeding values ranged from 0.956 to 1.015.

**Table 7:** Inflation of the estimated breeding values per model on the simulated dataset using three tests

	Moda	Modam	Coram	Corepe	Coramepe	se
TBV ~ EBV_reduced_	0.844	0.913	0.865	1.025	1.003	0.017–0.027
*y*_c_ ~ EBV_reduced_	0.967	0.980	0.983	0.993	0.998	0.018–0.027
EBV_full_ ~ EBV_reduced_	0.956	0.973	0.909	1.063	1.015	0.010–0.017

The inflations were estimated for each model. The first one is the slope of the regression of the true breeding values (TBV) on the predicted breeding values (EBV_reduced_). The second one is the slope of the regression of the phenotype corrected for fixed effects (*y*_c_) on the predicted breeding values (EBV_reduced_). The last one is the slope of the regression of predicted breeding values using the full dataset (EBV_full_) on the predicted breeding values using the reduced dataset (EBV_reduced_). The ranges of the standard errors (se) are presented in the last column. Moda is the model for which no maternal genetic effects are included and for which the direct-maternal environmental covariance is null. Modam is the model for which both the direct-maternal genetic and the direct-maternal environmental covariance are null. Coram is the model for which the direct-maternal genetic effect is considered as non-null while the direct-maternal environmental covariance is null. Corepe is the model for which the direct-maternal environmental effect is considered as non-null while the direct-maternal genetic covariance is null. Coramepe is the model for which both the direct-maternal genetic and the direct-maternal environmental covariance are non-null

To summarize, the breeding values showed no inflation when they were estimated with the Coramepe model. Depending on the response used, the other models ranked differently based on the inflation of breeding values. A slight inflation of the breeding values was observed when the direct-maternal environmental covariance was ignored. Considering only the direct-maternal genetic covariance led to great inflation of the breeding values. Dropping further components from the model led to severe inflation of the predicted breeding values.

## Discussion

### Feasibility of estimating the direct-maternal environmental correlation

Early attempts to account for the direct-maternal environmental correlation included regression of the offspring phenotype on the dam phenotype [7, 8], but this method results in a biased estimate of the correlation [7]. In 2006, Bijma showed [9] that it was, in theory, possible to implement a direct-maternal environmental correlation into the software that is used to predict breeding values. In 1997, Koerhuis and Thompson [1] estimated the direct-maternal environmental correlation using a method that is similar to that used here, although they used derivative-free REML methods, which generally tend to be numerically unstable for complex models. In 2015, Munilla and Cantet [11] proposed an approach to estimate the direct-maternal environmental correlation using a Bayesian inferential procedure. However, this method is computationally demanding, and may be difficult to apply to large datasets.

Here, we demonstrate a simple approach that can be easily implemented into standard software such as the DMU software [18] given that some of the pseudo-parameters in the model can be constrained to predetermined values. With our approach, it is possible to estimate the direct-maternal environmental correlation in models including all parameters. To fit this model using standard software, it is only necessary to add a dummy residual variance that is restricted to a constant as described in the "Methods" section. Compared with the Modam model, which includes both genetic and environmental maternal effects, but assuming them to be uncorrelated, adding both a direct-maternal genetic and an environmental covariance did not lead to an increase in computation time. As shown in our simulation, with this method we can accurately estimate the direct-maternal environmental covariance. Our simulation showed that adding the dummy residual to the model led to accurate estimates of the variance components. In addition to the possibility of estimating the direct-maternal environmental covariance, adding the dummy residual in the model can also facilitate their implementation, for example, to estimate the correlation between the environmental effect of production traits and litter traits in other species or to model the heterogeneous residual variance using standard software that did not necessarily offer such options.

### Relation between the direct-maternal genetic covariance and the direct-maternal environmental covariance

A negative correlation between the direct additive genetic effect and the maternal genetic effect has often been reported in the literature for different species [1, 2, 6, 7]. It has been proposed that such a negative direct-maternal genetic covariance was due to a highly negative environmental covariance [7, 8]. However, apart from specific cases, there is no reason to assume that the direct-maternal genetic covariance should be positive [5, 9]. Here, we found a positive correlation between environmental effects whereas the genetic correlation was negative, which is similar to results reported by Koerhuis and Thompson [1]. However, in their study, they did not observe differences in BW between males and females and considered them as a single trait, which is most likely due to the individuals in their study being older than ours. Indeed, it is well known that the maternal effects decrease with increasing age of the chicks [13, 17]. Koerhuis and Thompson [1] also reported a negative direct-maternal genetic correlation, which suggests that this is a general relation in chicken.

The negative Cor(a, m) observed here is not due to a negative direct-maternal environmental covariance that was ignored in some of the models. The main consequence of this negative Cor(a, m) would be that although the dams were selected for their higher genetic potential which is transmitted to offspring, it will have a negative impact on the maternal component of the genetic potential of their offspring. This negative direct-maternal genetic covariance may be explained by the fact that broilers are selected mainly on production traits, including their growth potential. It is well known that a high-level of production can have a negative effect on fitness and therefore on reproduction ability [21, 22]. Based on resource allocation theory [21], it is reasonable to assume that the heaviest dams will allocate more of the absorbed energy to growth traits than to reproduction traits, and thus the eggs, from such dams, may contain fewer nutrients for the developing embryos, which will negatively affect the early growth of the chicks. However, because these dams are larger they will produce larger eggs creating a favorable environment for the development of bigger chicks [23, 24]. This could partially explain the positive correlation between the dam’s temporal environmental effect and the permanent effect of the dam on her offspring, denoted as cor(e, pe). Nevertheless, it is uncertain whether the advantage conferred from hatching from a larger egg is maintained at a later age [23, 24]. However, it is also known that the permanent environmental maternal effect decreases as the chick grows [13], and thus it would be interesting to study how the cor(e, pe) changes as the chick grows. However, this could not be done with our data because BW was measured at a fixed age. The positive Cor(e, pe) could also be explained by effects that are not included in our model, such as non-additive genetic effects [9]. Most likely this correlation is the consequence of different factors rather than having one simple explanation. Developing a more complex model, which would allow the dissection of this so-called environmental covariance, by adding non-additive genetic effects, and external information such as microbiota (egg and dams), might help better understand this relationship.

### Assessing the predictive ability of models in the presence of maternal effects

In the presence of maternal effects, it is necessary to adopt a specific strategy of cross-validation [1, 12, 13]. Different approaches have been suggested such as estimating the breeding values using whole and partial datasets [12] or using half-sib correlations [13]. In our simulation study, we were not able to rank correctly the different models based simply on the correlation between the phenotypes corrected for the fixed effect and the predicted breeding value. As indicated by Legarra and Reverter [12], this might be because the different fixed effects are not estimated correctly, which results in inaccurate corrected phenotypes. However, in the simulation, we could use the true value of each of the fixed effects and thus obtain the true phenotype corrected for the fixed effects, but still we were not able to rank the models correctly. The main issue is that for all the models, except the Coramepe model, both predicted breeding values and *y*_c_ might be biased in the same direction by maternal effects, making validation of the model difficult. Based on the variance components, we can see that when the model changes, the definition of the predicted additive genetic effects will change. When the maternal genetic effect is ignored then the direct additive genetic effects capture part of these missing effects. Thus, the EBV obtained from the Moda model is different from that obtained with the Coramepe model. Indeed, with this model, the direct additive genetic effect did not capture some parts of maternal effects and of the direct-maternal covariance. Thus, because we compared models that change the definition of the additive genetic effects, the models are not compared on the same scale. For example, with the Moda model, which did not include any maternal genetic effect, the additive genetic variance was affected by the maternal genetic variance, when estimated from simulated data with a non-null maternal genetic effect. Thus, the EBV used to compute the accuracy is heavily biased by the maternal effects and the maternal effects are also included in *y*_c_ making the CV based on cor(*y*_c_, EBV_reduced_) problematic. In contrast, with the Coramepe model, it is possible to disentangle quite accurately the different elements. Thus, in this case, the EBV is not biased by the maternal additive genetic effect. Using the half-sib correlation, we were able to rank the models correctly in the simulation study although the differences were small. Thus, the power of this test for model comparisons is limited. The same is true for the correlation between the EBV_full_ and EBV_reduced_. The difference in predictive ability between the models was small. Nevertheless, when comparing models that might change the interpretation of the additive genetic or other random effects in the model, our simulation study highlighted the need to design the CV strategy carefully to be able to compare those models on the same base. Dividing the correlations by an adjusted heritability estimate based on the trait definition might provide different results; however, this can make their interpretation more complex because heritability estimates from mis-specified models might also be biased.

### Impact of the direct-maternal genetic and environmental covariances on predictive ability

In the simulation, accounting for direct-maternal genetic and/or environmental covariance led to breeding values with no significant inflation and was associated with a gain in accuracy compared with Moda. The main advantage of the models that include a direct-maternal genetic and/or a environmental covariance is, as expected, that they control the inflation of breeding values. It seems that environmental covariance has the biggest impact on the reduction of inflation of breeding values because adding the genetic covariance does not lead to further gain. In fact, adding the genetic covariance to the prediction model led to a slightly greater inflation of the breeding values, but it was not significant. This is because the permanent environmental effect is associated with the residual, that is, the components with the largest variance in all models.

Surprisingly, with real data, the LRT indicated a better fit using the Coramepe model, but the prediction of breeding values with this model led neither to an observable greater accuracy nor to a decrease in the inflation of breeding values compared with the Modam model. Because the LRT assesses the fit of a model with the given dataset, it does not assess the fit of this model to a new dataset as opposed to the CV strategy. Therefore, the Coramepe model is the model that fits our training population best, however, we are not able to confirm its superiority in the validation population. As discussed in the previous section, this might be because of the statistical test we used. However, we did not observe a reduction in the deflation of the breeding values. This absence of change in deflation might be due to the heterogeneous residual variance, which was neither simulated nor modeled, but which might be present, especially in the latest SR that constituted our validation population. Further model development, to account for residual heterogeneous variance and environmental covariance between the dam and its offspring is needed. In addition, other unknown events that might have occurred in the real data, were not considered. Identification of such effects could have resulted in a validation population that differed from the training population because of changes in diet or age at which BW was measured. Indeed, we observed that the estimates of some of the fixed effects presented a trend over SR and such a trend was also not considered in our simulation. A simpler explanation for this absence of gain in accuracy and reduction of deflation/inflation of the breeding values when using the complete model might be that in our population, environmental and direct-maternal genetic covariances are not important enough to impact the prediction of breeding values. Applying our method to other populations might help to investigate the impact of adding environmental and direct-maternal genetic covariances in other populations of broilers but also in different species.

### Breeding program

Considering the direct-maternal environmental covariance in prediction models led to a gain in prediction accuracy and a reduction of the inflation of the breeding values in the simulation. Thus, this may contribute to increasing genetic gain in the breeding program. Because of the negative direct-maternal genetic covariance, it might be interesting to select the dams not only on their direct additive genetic potential but also on their maternal genetic effect. In fact, with the Moda model, which does not consider the maternal genetic effect, the estimated breeding values capture a part of the maternal genetic effect, so that the individuals are already based on a composite EBV. Although the breeding values estimated with the different models were highly correlated (> 0.9), re-ranking among the candidates were observed (data not shown). Thus, depending on the model used to estimate the breeding values, different individuals will be selected to produce the next generation. We can also speculate on the consequences of ignoring the direct-maternal covariances for the breeding program, if these, in reality, are not significantly different from zero. To check the possible consequences of over-specified models, an extra simulation study was conducted for which the only difference compared to the simulation described in the "Methods" section was that both the genetic and direct-maternal environmental correlations were null. Therefore, if these are null, then the model becomes similar to a model that assumes no covariance, which results in no loss in predictive ability compared with the simplest model (see Additional file 1: Tables S1 and S2). One limitation to the correct estimation of the genetic parameter might be the number of generations in the data used. Adding both covariances did not lead to an increase in computation time (see Additional file 2: Table S3), which may be a critical point for practical breeding applications. Our analysis focused on BW, but this model could be easily extended to any traits that are affected by maternal effects.

## Conclusions

In this paper, we demonstrate a straightforward way to estimate the direct-maternal environmental correlation using standard software and to use it in models for the prediction of breeding values. This can be implemented without significant increases in computing time. The direct-maternal environmental correlation was positive while the direct-maternal genetic correlation was negative. Standard CV methods do not rank models correctly in the presence of maternal effects that are not included in models for genetic evaluation. The half-sib correlation and the correlation between EBV_full_ and EBV_reduced_ seem to be promising but we were not able to completely confirm the results obtained with the simulation studies when applied to real data, which indicates that there might be other unknown factors that influence BW and are not included in our analysis. In the simulation, we showed that when the direct-maternal environmental covariance is not null its consideration in model prediction will increase the accuracy of prediction and reduce inflation of breeding values, compared with a model that ignores it. Over-specifying the model by including covariances when in reality they did not exist did not lead to loss of accuracy. Therefore, implementing a model that includes the direct-maternal genetic and the direct-maternal environmental effects may lead to improved genetic gain, without impacting computation time in the routine genetic evaluations.

### Supplementary Information

**Additional file 1: Table S1.** Variance components estimated with Modam and Coramepe models and with the true model**. Table S2.** Predictive ability of the Coramepe and Modam models in the absence of genetic and environmental direct-maternal covariance**.** Additional file 1 provides information on the consequences of modeling direct-maternal genetic and environmental covariances when those covariances are null.

**Additional file 2: Table S3.** Computational time and convergence criteria for the five models using real data**.** This document provides details on the convergence criteria used and on the computational time needed for the five models.

## Data Availability

The data that support the findings of this study are available upon request from the corresponding author with permission from Cobb-Vantress.
