# Supplementary material for: Estimation and consequences of direct-maternal genetic and environmental covariances in models for genetic evaluation in broilers
Source: Genet Sel Evol. 2023 Aug 7;55:58. doi: 10.1186/s12711-023-00829-8 (PMC10405509; doi:10.1186/s12711-023-00829-8)
Supplement: Supplementary file 1 — Additional file 1: Table S1. Variance components estimated with Modam and Coramepe models and with the true model. Table S2. Predictive ability of the Coramepe and Modam models in the absence of genetic and environmental direct-maternal covariance. Additional file 1 provides information on the consequences of modeling direct-maternal genetic and environmental covariances when those covariances are null. [file 12711_2023_829_MOESM1_ESM.docx]

**Additional file 1**

**Simulation when genetic and environmental direct-maternal covariance are null**

In the same way as described in the Material and Methods section, we simulated a population but with null genetic and environmental direct-maternal covariance. In this case, the true model is then the model called Modam model. We then estimated the variance components and the breeding values using the Modam model and the Coramepe Model, as described before.

As shown in the table below presenting the variance components estimated with the two models and the true one, with the Coramepe model, the genetic and the environmental covariance are close to zero as expected.

**Additional file 1 Table S1**

**Variance components estimated with Modam and Coramepe models and with the true model**

|  | Modam | Coramepe | True VC |
| --- | --- | --- | --- |
| $\boldsymbol{\sigma}_{\boldsymbol{a}}^{\boldsymbol{2}}$ | 7982 (352) | 7884 (498) | 8046 |
| $\boldsymbol{\sigma}_{\boldsymbol{m}}^{\boldsymbol{2}}$ | 831 (138) | 837 (124) | 829 |
| $\boldsymbol{\sigma}_{\boldsymbol{pe}}^{\boldsymbol{2}}$ | 908 (78) | 909 (97) | 906 |
| $\boldsymbol{\sigma}_{\boldsymbol{e}}^{\boldsymbol{2}}$ | 19886 (168) | 19934 (257) | 19860 |
| $\boldsymbol{\sigma}_{\boldsymbol{am}}$ | 0 | 28 (208) | 0 |
| $\boldsymbol{\sigma}_{\boldsymbol{epe}}$ |  | 54 (198) | 0 |

Additive genetic variance ($\sigma_{a}^{2}$), maternal genetic variance ($\sigma_{m}^{2}$), environmental variance ($\sigma_{e}^{2}$), environmental permanent maternal variance ($\sigma_{pe}^{2}$), genetic, environmental covariances and correlations ($\sigma_{am}$, $\sigma_{epe}$, $r_{am}$, $r_{epe}$), with a simulated data where genetic and environmental covariance were null.

Modam is the model for which both the direct-maternal genetic and the direct-maternal environmental covariance are null. Coramepe is the model for which both the direct-maternal genetic and the direct-maternal environmental covariance are non-null. The True VC are the variance components used for the simulated population.

Standard errors of the estimates are in brackets.

In absence of genetic and environmental direct-maternal covariance, the Coramepe model becomes similar to the Modam model. This could be observed on the predictive ability, where identical accuracies of prediction and inflation of the breeding values were found.

**Additional file 1 Table S2**

**Predictive ability of the Coramepe and Modam models in the absence of genetic and environmental direct-maternal covariance**

|  | Modam | Coramepe | se |
| --- | --- | --- | --- |
| cor(TBV,EBV_reduced_) | 0.38 | 0.38 | 0.01 |
| TBV~EBV_reduced_ | 0.97 | 0.97 | 0.02 |

Observed accuracies of prediction are defined as the correlation between the true breeding values and the predicted breeding values (cor(TBV, EBV_reduced_)); The inflation was defined as the slope of the regression of the true breeding values (TBVs) on the predicted breeding values (EBV_reduced_).
Modam is the model for which both the direct-maternal genetic and the direct-maternal environmental covariance are null. Coramepe is the model for which both the direct-maternal genetic and the direct-maternal environmental covariance are non-null
