# Supplementary material for: Estimation and consequences of direct-maternal genetic and environmental covariances in models for genetic evaluation in broilers
Source: Genet Sel Evol. 2023 Aug 7;55:58. doi: 10.1186/s12711-023-00829-8 (PMC10405509; doi:10.1186/s12711-023-00829-8)
Supplement: Supplementary file 2 — Additional file 2: Table S3. Computational time and convergence criteria for the five models using real data. This document provides details on the convergence criteria used and on the computational time needed for the five models. [file 12711_2023_829_MOESM2_ESM.docx]

**Additional file 2: Table S3**

**Computing time and number of round to reach convergence for the five models using the real data**

|  | Moda | Modam | Coram | Corepe | Coramepe |
| --- | --- | --- | --- | --- | --- |
| Number of rounds | 24 | 26 | 28 | 24 | 25 |
| CPU time | 00:17:56 | 00:46:14 | 00:48:24 | 00:52:40 | 00:43:13 |

Number of round is the number of rounds needed to reach convergence criteria of 1E-11. CPU is the total computer time needed to estimate the variance components. Moda is the model for which no maternal genetic effects are included and for which the direct-maternal environmental covariance is null. Modam is the model for which both the direct-maternal genetic and the direct-maternal environmental covariance are null. Coram is the model for which the direct-maternal genetic effect is considered as non-null while the direct-maternal environmental covariance is null. Corepe is the model for which the direct-maternal environmental effect is considered as non-null while the direct-maternal genetic covariance is null. Coramepe is the model for which both the direct-maternal genetic and the direct-maternal environmental covariance are non-null.

Coram model needed more rounds to converge. Adding both genetic and environmental direct-maternal covariance to the model reduced the total CPU time.
